# Exploring Medical Students’ Perceptions of Near-peer Teaching in Musculoskeletal OSCE Preparation

**DOI:** 10.1007/s40670-026-02805-5

**Published:** 2026-06-26

**Authors:** David Wong, Ethan Allen, Yitong Li, Terese Bird, Jitendra Mangwani

**Affiliations:** 1 Leicester Medical School, University of Leicester, Leicester, United Kingdom; 2 Department of Trauma and Orthopaedics, University Hospitals of Leicester NHS Trust, Leicester, United Kingdom

**Keywords:** Near-peer teaching, OSCE, Musculoskeletal, Competency, Medical student

## Abstract

**Background:**

Earlier studies have demonstrated suboptimal confidence among recently graduated medical students in diagnosing and managing musculoskeletal (MSK) conditions, despite the high prevalence of MSK disorders, underscoring gaps in MSK education. As a result, Objective Structured Clinical Examinations (OSCEs) in MSK medicine can be particularly challenging for novice medical students. Near-peer teaching (NPT) may be a valuable teaching modality for improving medical students’ knowledge and confidence.

**Objective:**

To explore medical students’ perceptions and the factors influencing their perceived value of NPT, specifically for MSK OSCE preparation.

**Methods:**

Students enrolled in the Bachelor of Medicine and Bachelor of Surgery (MBChB) programme at the University of Leicester who had previously received MSK OSCE-related teaching from a near-peer tutor participated in semi-structured interviews. Participant recruitment ceased after interviews were conducted with 12 participants, as the research team judged that the interviews provided sufficient information power to develop a rich and nuanced thematic analysis to address the research question.

**Results:**

Interviews with 12 participants yielded four themes: the learning environment, exam-focused guidance, the attributes of the near-peer tutor, and the logistics of delivery. Students perceived NPT as a safe learning environment and a source of OSCE-specific insights from tutors who had recently and successfully navigated the same assessments, which is not easily recreated in faculty-led teaching. Tutor attributes, such as relatability, approachability, and their perceived commitment and dedication, emerged as key determinants of favourable perceptions of NPT. However, the extracurricular nature of NPT offered flexibility for some, while others felt it added additional time constraints when faculty-led teaching was easier to access. Students suggested integrating NPT into the curriculum to improve participation.

**Conclusion:**

NPT was perceived as a highly valuable learning modality for developing MSK-related competency for students preparing for their OSCEs.

**Supplementary Information:**

The online version contains supplementary material available at https://doi.org/10.1007/s40670-026-02805-5.

## Background

Musculoskeletal (MSK) conditions are among the most common reasons patients seek medical care [1]. Therefore, competent diagnosis and management of these conditions is crucial for physicians. However, a notable gap exists between this clinical need and the confidence of recently graduated medical students. For example, only 40% of recently graduated medical students from the United Kingdom consider themselves competent in MSK medicine [2]. A more recent study reported lower students’ confidence in orthopaedic surgery among Canadian medical students in their 2nd, 3rd, or 4th year of study than in other specialities, such as general surgery, obstetrics and gynaecology, cardiology, respiratory medicine, gastroenterology, and psychiatry, underscoring gaps in MSK education [3]. In addition, the study found that over 60% of students retained only 30–59% of orthopaedic knowledge from prior coursework [3]. As a result, Objective Structured Clinical Examinations (OSCEs), which form a fundamental component of summative assessment for medical students worldwide [4], can be particularly challenging in MSK medicine due to the complexity of MSK disorders and the practical skills required to diagnose and manage them accurately.

Several factors have been proposed to explain the suboptimal confidence in MSK medicine among undergraduate students, such as limited exposure to dedicated MSK curricula during clinical training [5], limited availability of clinical musculoskeletal internships [6], and inadequate quality of MSK education [7], highlighting gaps in medical training. Although addressing these gaps will require comprehensive curricular transformations focused on the quality and depth of MSK education, existing systemic barriers, such as curriculum overcrowding, a lack of specialised faculty, and limited clinical placement capacity, hinder such transformations [5–11]. As a result, intervention programs comprising supplementary coursework focused on MSK medicine, MSK simulations, podcasts, hands-on training with prosections, flipped classrooms, and peer evaluation have been employed to address this gap, but have largely focused on interns and residents in physical medicine, rehabilitation, and emergency departments and have yielded mixed results [6, 7, 12, 13].

Near-peer teaching (NPT), where a senior student (a ‘near-peer tutor’), typically with at least one academic year ahead in their training, teaches a junior student within the same educational program [14, 15], is emerging as an effective adjunct modality to improve the MSK-related knowledge of medical students, especially in the context of clinical examination skills [16, 17]. NPT has been shown to benefit both tutors and tutees by improving their knowledge and skills, thereby facilitating their progress towards becoming competent physicians [18, 19]. Although many NPT programmes, especially those that are student-led, are extracurricular and delivered in an informal setting, integrating them into the formal curriculum is generally considered more efficacious in the long term [17, 20–22]. NPT programmes have also gained recognition and popularity in medical education, with many medical schools incorporating NPT into their curricula [23–25]. Blake [26] reported that practising with peers was the second most preferred method for learning MSK clinical examination skills, thereby supporting the use of NPT specifically for developing competency in MSK medicine.

Nonetheless, there is a paucity of studies examining the effectiveness of NPT for MSK OSCEs and its perceived value in preparing for these OSCEs. Therefore, the current study explored medical students’ perceptions and the factors influencing their perceived value of NPT, specifically for MSK OSCE preparation, with the aim of informing future efforts to develop standardised approaches to optimise medical students’ preparation for MSK OSCEs.

## Methods

### Study Design

A descriptive qualitative study design was chosen to facilitate an in-depth exploration of medical students’ experiences and perceptions [27, 28]. A semi-structured interview was selected as the data collection method because it provides a structured framework and allows the researcher to thoroughly explore participants’ responses through follow-up questions [28]. Ethical approval was obtained from the University of Leicester Research Ethics Committee before commencing participant recruitment and data collection.

### Participants

Participants were invited to our study between March and April 2025 via the email service integrated in our learning management system (Blackboard) or through relevant student societies. DW was primarily responsible for distributing invitation emails via Blackboard and for formally contacting student societies via email. Two members of the research team (DW and EA) held committee positions within one of the societies used for recruitment. To mitigate any potential conflict of interest, recruitment was conducted using standardised email invitations and explicitly stated that participation was entirely voluntary and that non-participation would not affect students in any way. Individual society members were not directly approached by the researchers regarding participation decisions.

The inclusion criteria for participants were students enrolled in the Bachelor of Medicine and Bachelor of Surgery (MBChB) programme at the University of Leicester who had previously participated in at least one MSK OSCE-related teaching delivered by a near-peer tutor during their medical studies. As students in their 2nd year of medical education and above can undertake MSK OSCE, all students in their 2nd year through to 5th year were eligible to participate in this study. The NPT attended by the participants was not a single, standardised NPT programme. Rather, the NPT sessions attended were extracurricular, student-led, and varied in structure, timing and content.

All eligible participants received a participant information sheet, privacy notice, and consent form. The participants were reminded of the voluntary nature of their participation and their right to withdraw from the study at any time without consequences. They were also informed that the interviews would last approximately 30 to 60 min and would be kept confidential, with all transcripts anonymised for analysis. Therefore, near-peer tutors would not know that their peers have participated in the study, have access to their responses, or be able to identify individual participants. All participants signed the consent form agreeing to participate in the study and had their interviews audio-recorded, with the knowledge that their de-identified quotes may be included in publications.

### Data Collection

The interview guide (Supplementary Information 1) was collaboratively developed by the research team using a semi-structured qualitative approach [29]. It consisted of closed questions to guide the discussion and open-ended questions to encourage deeper discussion. Participants were asked about their experiences with NPT in MSK OSCE preparation, opinions on how it might be incorporated into timetabled university teaching, what they enjoyed and found worked well, and what they thought could be improved in the future.

Semi-structured interviews were conducted either in person at the University of Leicester or online via Microsoft Teams in a relaxed manner, allowing for open discussion and flexible exploration of participants’ perceptions. Ten interviews were conducted face-to-face, and two via Microsoft Teams. Each interview was conducted individually. All interviews were facilitated by DW, with EA attending three of the twelve interviews. EA attended only three interviews due to time constraints unrelated to this study. Both DW and EA were in their fourth year of medical school at the time of conducting the interviews.

Participant recruitment ceased after interviews were conducted with 12 participants, as the research team judged that the interviews provided sufficient information power to develop a rich and nuanced thematic analysis to address the research question. This judgement was based on the study’s aim, the relatively homogeneous sample and the depth of participants’ responses.

### Data Analysis

All interviews were audio-recorded and transcribed verbatim by DW and EA. All recordings were listened to, and transcripts read multiple times by DW and EA to fully immerse themselves in the data. Subsequently, the transcripts were analysed using the reflexive thematic analysis framework developed by Braun and Clarke [30]. This process allowed for the identification of recurring patterns and themes in participants’ experiences. Initial codes were generated for meaningful sentences, and then similar codes were grouped into potential subthemes. These subthemes were reviewed and refined into overarching themes that captured the most meaningful patterns in participants’ experiences and perceptions. Throughout the analysis, regular meetings were held to discuss coding interpretations and thematic patterns. Any discrepancies were resolved by DW and EA through reflexive discussion to deepen analytical insights and ensure agreement on the final themes.

## Results

Of the 12 participants interviewed, eight (67%) were in Year 2, three (25%) in Year 3, and one (8%) in Year 4 of the five-year MBChB programme. Seven (58%) of the participants identified as female, and five (42%) identified as male. Ages ranged from 18 to 23 years, with a mean age of 20 years. The interviews ranged in length from 28 to 52 min, with a mean of 41 min. After reviewing the transcripts of these interviews and eliminating duplicates, we identified 87 codes, which were collated into four themes and nine subthemes. A summary of the identified themes and subthemes is presented in Table 1.

**Table 1:** Themes and subthemes identified using thematic analysis

Themes	Subthemes
The learning environment	Importance of a low-stakes environment
	Negative consequences of a high-pressure environment
Exam-focused guidance	Importance of strategic insight and exam relevance
	Importance of contextualisation through demonstration
Attributes of the near-peer tutor	Importance of relatability
	Importance of approachability
	Importance of perceived commitment and dedication
The logistics of delivering NPT	Importance of extracurricular flexibility
	Importance of consistency and standardisation

### The Learning Environment

#### Importance of a Low-stakes Environment

The informal, relaxed nature of NPT was perceived as a key factor in its effectiveness. Compared with more formal teaching settings, this low-stakes environment allows students to practise without fear of judgement. As one student explained:

*“NPT is less intimidating than traditional university teaching because I think you’re less worried about getting things wrong or making a mistake as you’re being taught by somebody who’s literally been in the same position as you were very recently. There was no pressure to get things right*,* and this definitely made me more open and willing to participate” *(*Participant 6*)*.*

The participants also noted that this supportive environment promoted inclusivity and engagement as exemplified by the following quote from one of the participants:

*“Everyone got to have a go to practise*,* everyone got feedback*,* and no one felt scared about asking questions or taking part; it was very open and welcoming” *(*Participant 3*).

Moreover, students highlighted the psychological safety of NPT:

*“The more relaxed environment [of NPT] meant you’re less likely to be sort of ashamed or embarrassed if you made a mistake. I find it really useful because it gives you a safe space; it’s not like you’re being examined or judged” *(*Participant 5*).

#### Negative Consequences of a High-pressure Environment

In contrast, the formal setting of faculty-led teaching, when used for MSK OSCE preparation, increased anxiety and hindered participation, such as students volunteering to practise and ask questions. Five medical students described feeling intimidated by the presence of the faculty tutor and unfamiliar peers. As one student reflected:

*“[Faculty-led teaching] is done in front of the rest of your study group where there’s seven or eight students there*,* who you might be shy in front of and the tutors who will potentially be marking my exam when it comes to it. Whereas*,* in [NPT]*,* if I do have any like worries or questions*,* I’m more likely to ask it” *(*Participant 7*).

Another student explained how the increased anxiety actually prevented them from wanting to participate in the MSK examination practice:

*“[Faculty-led teaching] is more nerve-wracking*,* especially when it’s like a GP [tutor] watching you*,* and they know exactly what you should do… I didn’t want to volunteer myself [to practise the MSK examination] because of that”* (*Participant 3*).

One student contrasted the two dynamics of NPT and faculty-led teaching. They perceived how having a senior faculty tutor led to a higher expectation to perform and therefore to performance anxiety:

*“With the near-peer teachers*,* it’s easier to make mistakes because they are also students… whereas with the [faculty-led] tutors*,* it becomes a way of like ‘oh we taught you this*,* how do you not know this…’ Sometimes it’s just too stressful for me” *(*Participant 8*).

### Exam-focused Guidance

#### Importance of Strategic Insight and Exam Relevance

Ten participants perceived NPT as highly relevant to their MSK OSCE preparation and learning. They valued the strategic insights for preparing for the OSCE provided by tutors who had recently prepared and successfully navigated the same assessments. This knowledge, they felt, was extremely valuable and not easily recreated through faculty-led teaching. As one student explained:

*“I think it’s really valuable to have a student’s [near-peer tutor] point of view on how best to conduct an examination*,* what’s worked best for them*,* and how they found the whole OSCE experience and how best to prepare for it”* (*Participant 7*).

Another student explained that the near-peer tutors were more likely to have recently taken the same OSCEs and could provide more specific, practical guidance aimed at helping students prepare for OSCEs, while remaining concise:

*“With the advice given by the near-peer teachers*,* it felt more relevant as they had recently sat the OSCE exams and so gave tips and tricks on how to succeed*,* which were really helpful and something that you can’t get from an employed tutor by the university. It was really useful as it cut out the waffle; I sometimes find in university teaching and get straight to the point of what you need to know”* (*Participant 2*).

#### Importance of Contextualisation Through Demonstration

In addition to strategic knowledge, participants highlighted how NPT enabled them to contextualise specific special tests and findings in MSK OSCEs. Near-peer tutors would provide relevant advice on how to improve their communication with patients to avoid misunderstanding and demonstrate physical tests that aid memory. As one student explained:

*“They were teaching us how to phrase things for the exam. When I’m practising with my friends and speaking with them*,* they know what I’m implying*,* so they just go with it. Whereas when we were doing it with the near-peer tutors*,* they were saying ‘oh*,* you want to be more clear; this is how I would phrase it because otherwise the patient would do so and so.'…Special tests for the shoulder exam weren’t explained that well*,* but the tutors explained how to perform them*,* what a positive finding would look like*,* and the pathophysiology behind them*,* which helped me remember them in my OSCEs”* (*Participant 3*).

One student valued how near-peer tutors drew on their recent experiences to illustrate the unpredictable nature of OSCEs. They demonstrated clinical findings such as a limp or patient wince, which allowed learning to move beyond a static checklist:

*“I like how the [near-peer] tutors talked about their OSCE experiences and acted out what different limps would look like in a gait*,* or how a patient would react when you’re examining them”* (*Participant 11*).

This approach, as another student highlighted, contextualises physical signs and better prepares for the interactive elements of an OSCE:

*“That helped me kind of put things into perspective as to what I may encounter in an OSCE or even a real scenario…I really struggled with remembering passive movements*,* but the tutors gave us useful ways to remember how to perform them”* (*Participant 10*).

### Tutor Attributes

Along with the voluntary nature of NPT, which was perceived as a commitment and dedication on the part of the near-peer tutors, relatability (i.e., participants relating to the tutor’s background and experiences) and approachability (i.e., participants finding it easy to approach and talk to the tutor) emerged as important tutor attributes. While relatability and approachability are closely linked and often overlap, the two attributes are considered distinct constructs in empirical research [31–33]. Therefore, participants’ statements related to relatability and approachability were coded and presented as separate subthemes.

### Importance of Relatability

Throughout the participants’ interviews, students underscored the relatability between themselves and the near-peer tutor as an important factor in their MSK OSCE preparation, especially in understanding complex topics more easily and engaging more actively with the learning. As one student highlighted:

*“With [near-peer] students*,* you find a way to digest information in a more understandable fashion… [the near-peer tutors] have found their own ways to really understand the content*,* and then when they teach it to you*,* it’s more understandable for you as well*” (*Participant 4*).

Another student mentioned that near-peers can empathise more easily with them, creating a more inspiring atmosphere:

*“I liked the encouragement I received from the older [near-peer tutor] students; that was also quite amazing and made me feel much more confident. I think*,* as they’ve been through the same process as us in the past*,* they can relate with us and understand the stress and difficulties in practising for MSK OSCEs” *(*Participant 11*).

Ultimately, the near-peer tutor-tutee relationship emerged as an important factor in effective learning, breaking down the initial awkwardness barrier and accelerating the learning process. One student emphasised:

*“[The] main benefit of [NPT] is that it’s a lot easier to relate. I feel like there’s a kind of automatic rapport*,* especially for OSCE-based stuff*,* where it’s all speech-based*,* and rapport is kind of important. I feel like it’s a lot easier to get a baseline of where everyone’s more engaged*,* and you’re taking more away from it than*,* say*,* doing OSCE practice with a lecturer*,* or a Clinical Teaching Fellow [faculty tutor]*,* that I have never met before*,* and there’s a lot of time where you might spend trying to figure out each other” *(*Participant 12*).

### Importance of Approachability

Participants reported feeling more comfortable seeking clarification and admitting gaps in their knowledge because they perceived the near-peer tutors as more approachable. This reduced intimidation factor was seen as crucial for creating an effective learning environment. For example, one student stated:

*“I think because they’re more approachable*,* it is easier to raise concerns or things you’re finding difficult*,* so it improves the experience and makes it feel less intimidating” *(*Participant 1*).

Another student reinforced this dynamic:

*“Near-peer tutors were definitely more approachable than [faculty tutors]*,* and it was a bit more daunting asking [faculty tutors] questions*,* as they seem quite intimidating*,* and it felt like there was this power imbalance. However*,* during the near-peer teaching*,* I felt much more confident because there wasn’t that power imbalance.” *(*Participant 3*).

This approachability also manifested as greater flexibility towards different teaching styles. Near-peer tutors were able to adapt their teaching methods to meet individual student needs, creating more personalised learning experiences. It brought together different ways of thinking to collaborate and help one another learn. These experiences are what stick in a student’s mind when they revise and answer exam questions:

*“I find [NPT] a lot more valuable*,* and [near-peer tutors] are a lot more approachable and relatable. [Faculty tutors’] way of teaching might not necessarily be the way that you learn*,* but with the near-peer tutors*,* I find that people are a lot more accommodating to different learning styles*,* and so it’s easier to find someone who helps your learning than when it’s just with your given university tutor” *(*Participant 7*).

### Importance of Perceived Commitment and Dedication

The participants also recognised that the voluntary nature of NPT contributed to the quality of teaching. As most NPT is extracurricular and tutors volunteer to teach, this was seen as an indicator of genuine commitment and teaching dedication. One student expressed this as follows:

*“An upside of [NPT] is that most of the tutors and students are volunteering to do it. And if people are wanting to do it*,* then they’re going to put their own time and energy into it*,* and they’re going to do the best job that they can…there’s no kind of ulterior motive*,* or they’re not being forced to do it. So*,* I feel like that is maybe like the best motivator that gets good teaching” *(*Participant 4*).

### The Logistics of Delivering NPT

#### Importance of Extracurricular Flexibility

For all the participants interviewed in this study, NPT for MSK OSCE preparation was extracurricular, and no curriculum-integrated activities were provided. Some students perceived this as an advantage, while others saw it as a disadvantage. For example, as one student explained:

*“I enjoyed [NPT] because of the freedom it offers you with when you would do it*,* how you would do it*,* what you would do within it and stuff like that*” (*Participant 11*).

On the other hand, another student contrasts this with the difficulty of finding time for NPT:

*“It is an effort to attend an NPT programme*,* as it’s out of your own time… it is a lot of effort versus just going to a university-led teaching session which has already been scheduled into your timetable for you” *(*Participant 4*).

One student perceives NPT’s extracurricular aspect as a benefit due to flexibility, while also arguing that if mandated, more people would attend NPT:

*“It’s your decision whether you choose to participate or not*,* which I think is a great thing because you can attend when you feel like it; it means people who attend actually want to learn and engage more… If it was scheduled in the official timetable*,* I guess that people will be more likely to attend and also more people will be able to attend it too” *(*Participant 8*).

#### Importance of Consistency and Standardisation

According to the students, the most important way to standardise the teaching was to ensure it aligned with what they would be marked on in their exams. One participant described how this was appreciated by them, saying:

*“I think it’s important that [NPT] comes from students that have the OSCE mark scheme in front of them*,* one way of making sure it was accurate… I feel like it’s kind of a more consistent and mark-scheme-approved kind of way of doing [NPT]” *(*Participant 4*).

Another aspect of the teaching that requires standardisation is ensuring that near-peer tutors have excelled in their exams so they can teach and answer students’ questions generated during the teaching session:

*“Each tutor needs to be confident and have a good knowledge of the MSK examinations and pathology to be able to teach us and answer students’ questions. I guess knowing that the tutors have done well in their OSCEs would help with making sure that the teaching was of good quality” *(*Participant 6*).

However, the same student went on to explain that they appreciated the tutors’ individual experiences and how they could be used to offer advice:

*“I don’t think there’s actually that much proper training that’s needed for NPT because I think the benefit of [NPT] is the fact that it’s a bit more informal… like ‘yeah*,* I was in your position like a year ago*,* this is what I did’… It’s based more on individual experience rather than giving everybody the same kind of training like for university teaching*,* because otherwise*,* then you’re taking away that individual aspect of it*,* which I think is a massive benefit” *(*Participant 6*).

## Discussion

The current study aimed to explore medical students’ perceptions of NPT for MSK OSCE preparation and the factors influencing the perceived value of NPT. Thematic analysis of 12 interviews with undergraduate students preparing for OSCEs who had previously attended at least one NPT yielded four key themes: the learning environment, exam-focused guidance, the qualities of the near-peer tutor, and the logistics of delivery. Participants valued the relaxed, low-stakes, and supportive environment of NPT. By contrast, faculty-led MSK teaching was often perceived as intimidating, and respondents felt embarrassed to participate out of fear of judgment. This finding is consistent with Edmondson’s concept of psychological safety, which emphasises the importance of an atmosphere in which learners can make mistakes without the fear of negative consequences [34]. The perception of the psychological safety fostered by NPT for MSK OSCE preparation among our participants has also been observed in the broader field of medical education. Safe learning environments have been associated with higher engagement and lower anxiety than learning in unsafe settings [35–37].

Participants identified several qualities of near-peer tutors that actively fostered psychological safety and reinforced the community of practice essential for effective learning. The value students placed on relatable role models who understood their challenges and conveyed material in accessible language aligns with previous reports [14]. In contrast, senior teachers commonly have an expert blind spot, which can lead them to struggle to understand students’ challenges because their own knowledge and problem-solving processes have become automatic and subconscious [38, 39]. As near-peer tutors had recently completed the same OSCEs, they were able to teach at a level more closely aligned with their tutees than more senior tutors, which enabled them to provide clear, concise information, free of jargon, to help contextualise the challenging aspects of MSK examinations. As a result, participants also perceived near-peer tutors as more credible sources of practical, experience-based strategies to address challenges in MSK OSCEs and highly valued the exam-focused guidance from NPT. Participants explained that being able to relate to their near-peer tutors enabled them to understand information more easily and to create a more encouraging learning environment, which improved their confidence. Several studies have previously observed that relatability enhances students’ confidence and self-efficacy, and therefore their cognitive engagement with the subject [40, 41], albeit not specifically in the context of MSK OSCE preparation.

Approachability of the near-peer tutor was another key quality perceived by the participants. Participants felt that near-peer tutors were more approachable than more senior faculty tutors, particularly for asking questions or seeking clarification. Breaking down this power hierarchy between teachers and students, which is commonly seen in traditional medical education, facilitates more open learner‒tutor interactions and encourages active participation, thereby allowing learners to be more confident in themselves [42]. Similarly, the participants identified the near-peer tutors’ voluntary motivation as a notable quality. The perception that tutors were enthusiastic volunteers, rather than conscripts, was seen as a key indicator of their enthusiasm and a major contributor to the overall positive reception of the NPT. This aligns with existing evidence that a teacher’s enthusiasm positively influences student engagement and commitment to their learning [41].

Collectively, the relatability, approachability, and intrinsic motivation of near-peer tutors as key determinants of favourable perceptions of NPT strongly align with the theory of situated learning proposed by Lave and Wenger [43], which posits learning as a social process where novices begin at the periphery of a community’s practice by engaging in simple and low risk but meaningful tasks (legitimate peripheral participation), gradually progressing towards fuller participation in a scaffolded manner rather than the acquisition of decontextualised knowledge. Near-peer tutors who have previously navigated the MSK OSCE preparation and examination come from the same learning community as novice participants and are closer to the novice learners in terms of perspective and experience than formal teaching staff [21, 44]. As a result, near-peer tutors may be better situated to provide legitimate peripheral participation and timely scaffolding in line with the learner’s zone of proximal development [21, 45]. However, the extracurricular nature of NPT was seen as both a strength and a stumbling block for accessing content. While many appreciated the flexibility it provided and how scheduling studying into their free time made them feel productive, others felt that attendance would mean going out of their way when faculty-led teaching was easier to access.

Discussions with participants on standardising how near-peer tutors teach offer important insights. While it is important that students can answer their questions and receive a largely similar experience, the benefits of individuality and drawing on one’s own experience cannot be overlooked. As previously described, one of the primary motivations for students attending NPT is to gain insider knowledge and to learn how the tutors succeeded in the same exams that the students are currently preparing for. By rigidly standardising the passage of this knowledge, many ways of achieving success and of approaching the same problem from different perspectives would be lost. Therefore, collecting these ideas and experiences before teaching is ideal so they can be shared with all the tutees.

Our primary observation that students perceive NPT as highly valuable for MSK OSCE preparation aligns with prior research on NPT’s acceptability and effectiveness in the broader context of medical education. Program evaluation studies indicate that NPT provided a safe and comfortable environment for tutees, while offering constructive feedback and role modelling [19, 35, 46]. Echoing these findings, a systematic review of 16 studies found that students were more comfortable in NPT scenarios than in expert-led or faculty-led teaching, and that NPT was at least as effective as expert teaching groups or faculty-led teaching, but flagged potential concerns about the teaching proficiency and knowledge of near-peer tutors [47]. Participants in the current study also highlighted the need for high proficiency and knowledge among near-peer tutors, although they did not explicitly report encountering near-peer tutors with poor proficiency or knowledge. In addition, some authors have argued that integrating NPT into the curriculum could enhance the development of medical students’ professional identity [21]. Interestingly, studies show that the benefits of NPT extend beyond tutees; near-peer tutors achieve higher exam scores with fewer passive semesters than non-tutors [48]. The positive impact of NPT on tutors may be mediated by the consolidation of knowledge, teaching, and facilitation skills, as well as by greater confidence after serving as a near-peer tutor [46].

### Limitations and Future Directions

While this is the first qualitative study to use semi-structured interviews to explore medical students’ perceptions of NPT in the context of MSK OSCE preparation, it is not without limitations. First, although purposeful sampling was used to recruit students from all years of study, the majority of participants were in their second year, with underrepresentation from years 3 to 5. This sampling bias may have occurred because the interviews were conducted from March to April, when years 3 to 5 students had clinical placements and may have been less inclined to give up time for an interview. In addition, our study reflects the perceptions of self-selected participants and therefore does not capture the perceptions of students who did not engage with NPT. Thus, it is possible that including the NPT-related experiences of students in years 3 to 5, or of those who did not engage with NPT, could have yielded additional insights.

Second, this study was conducted at a single medical school, which may limit the generalisability of the findings. Nevertheless, the transferability of the results to other similar medical education contexts was maintained [40]. Third, because the NPT sessions attended by participants were not standardised or formally documented, we cannot describe a single, replicable educational intervention. Our findings reflect students’ perceptions of NPT in various informal formats. Lastly, all interviews were conducted by researchers from the same institution, which may have introduced social desirability bias. However, the advantage of this approach is that, as insiders, the interviewers were able to understand nuances and niches specific to medical school [49]. In addition, we explicitly encouraged critical feedback during the interviews to mitigate this bias.

It is worth noting that despite the high perceived value of NPT, the current study was not designed to evaluate whether participants who attend NPT for OSCE preparation achieve better outcomes than those who prepare using other modalities, such as self‑study or faculty-led tutorials. Although randomised controlled trials indicate small‑to‑moderate improvements in academic outcomes among medical students, especially clinical/practical skills and short‑term exam performance with NPT compared to faculty-led tutorials [50–52], the impact of NPT on OSCE outcomes has not yet been reported and warrants further investigation before any formal integration into MSK OSCE preparation.

Despite these limitations, our findings build on existing literature, adding further nuance to the understanding that the perceived value of NPT is multifaceted, thereby providing valuable insights for medical educators in developing and reforming their NPT initiatives for MSK OSCE preparation. First, educators and researchers are encouraged to pilot and evaluate different models to integrate NPT into MSK OSCE preparation during undergraduate medical education, such as a hybrid model of NPT and formal faculty, to determine the most effective ways to ensure that the learning objectives are met without compromising the relaxed, low-stakes, and supportive environment of NPT, as well as the reliability and approachability of near-peer tutors, highly valued by the participants. Future NPT programmes could integrate sessions into the formal timetable, allowing for greater access and removing the barrier of students having to sacrifice personal study time or clinical placement hours. This curricular integration would ensure that all students, regardless of their external commitments or confidence levels, have the opportunity to benefit from NPT. Additionally, rotating attendance could be implemented to ensure equitable exposure across the entire student cohort, rather than relying on self-selection, which may disproportionately favour already confident or engaged students.

Second, future studies could use structured discussions, such as focus group interviews, among students, faculty, and near-peer tutors to further explore students’ preferences for NPT and how best to integrate it into medical curricula. Such dialogues would not only help faculty reflect on their teaching practices but also highlight how institutional and regulatory rules might shape formal teaching approaches, in contrast to the more flexible methods of near-peer tutors who operate outside these constraints.

Lastly, it would be valuable to determine whether the benefits of psychological safety and exam-specific guidance reported by participants in the current study for MSK OSCE preparation translate to other clinical domains that require physical examinations, such as neurology or cardiology. Collectively, the findings from such initiatives will help inform the development of best practice guidelines for sustainable NPT programmes.

## Conclusions

Medical students shared positive experiences with NPT across four main themes—comfortable learning environment, focus on exam-specific guidance, near-peer tutor attributes, and the flexibility of this teaching style—and expressed eagerness to see it delivered on a broader scale. Students explained how a safe learning environment led them to contribute more than they would in faculty-led teaching and how this resulted in them gaining more from this style of teaching. Thus, participants perceived NPT as a highly valuable and effective learning modality for improving MSK-related confidence as they prepare for their OSCEs. Given this finding, along with previous literature on the benefits of NPT, medical educators may find NPT beneficial for MSK OSCE preparation in their own institutions.

## Supplementary Information

Below is the link to the electronic supplementary material.

Supplementary File 1 (DOCX 25.2 KB)

## Data Availability

The datasets generated during and/or analysed during the current study are available from the corresponding author on reasonable request.
