# Supplementary material for: Exploring Medical Students’ Perceptions of Near-peer Teaching in Musculoskeletal OSCE Preparation
Source: Med Sci Educ. 2026 Jun 26;36(4):1797–807. doi: 10.1007/s40670-026-02805-5 (PMC13582695; doi:10.1007/s40670-026-02805-5)
Supplement: Supplementary file 1 [file 40670_2026_2805_MOESM1_ESM.docx]

**Interview Guide: Exploring Medical Student Perceptions of Near-Peer Teaching for MSK OSCE Preparation**

**Part A: Introduction**

- **Welcome and Introduction:**
  - Introduce yourself and the purpose of the interview.
  - Explain the research project and its significance.
  - Reiterate to the student that there are no right or wrong answers and that their honest opinions are valuable.
  - Ensure consent form has been signed and filled out by the participant.
  - Reassure them of confidentiality and anonymity of their responses.
- “Thank you for agreeing to participate. My name is [Name], and I am conducting this interview as part of a research study exploring student experiences with MSK OSCE preparation. Your participation is voluntary and you may withdraw at any time.

All responses will be anonymised and kept confidential. There are no right or wrong answers, I am interested in your personal perspectives and experiences. Any opinions which you mention will not be available to anyone apart from the research team. Do you have any questions before we begin?

To start off, please could you tell me a little about your overall experience with MSK OSCEs so far?”

**Part B: Core Questions**

| Section | Core Questions | Optional Probes |
| --- | --- | --- |
| Context and Exposure | 1. How would you define "near-peer teaching" in your own words?   *(Clarify only if needed)*: For this interview, we are defining it as a scenario where a more senior student teaches a more junior student.   2. Can you tell me about the near-peer teaching you have attended? 3. How do you typically approach MSK OSCE revision? | - Could you talk me through the format and structure of the near-peer teaching sessions? - Could you talk me through your revision schedule for MSK OSCEs? |
| Perceptions and Comparisons | 4. Can you tell me about your experiences of near-peer teaching for MSK OSCE preparation? 5. What do you think made near-peer teaching effective or ineffective for MSK OSCE preparation? 6. In what ways, if any, did participating in near-peer teaching influence your MSK OSCE preparation? 7. If you have experienced both, how would you compare near-peer teaching with teaching from faculty members or clinical instructors? | - How did you feel about those experiences? - How did the way they covered the material compare with other revision resources or teaching you’ve had? - Did you receive any feedback or marking during these sessions? If so, what were your impressions? |
| Interactions and Dynamics | 8. How would you describe the way you interacted with near-peer teachers? 9. Thinking about the near-peer tutors you've encountered, what characteristics stood out to you? 10. How did the group dynamic, if any, affect your experience of the sessions? | - Were any of these characteristics particularly helpful or unhelpful from the near-peer tutors? - How did these characteristics influence your experience? - Is there a particular interaction with one of the near-peers that really sticks out in your mind? - Did who attended the session influence your experience in any way? |
| Limitations | 11. What limitations or challenges, if any, have you observed with these sessions? 12. What factors might influence a student's decision to participate in, or refrain from, these sessions? | - Was there anything that made it harder to attend near-peer teaching sessions? - Were there any factors that affected your willingness to attend? - Are there any specific areas of MSK OSCE preparation where you feel near-peer teaching is less effective? |
| Future Directions | 13. What suggestions, if any, do you have for improving these sessions? 14. What role, if any, do you think near-peer teaching should have within the medical curriculum? 15. What format and frequency do you think would be most effective for these sessions? | - What kind of support or training do you think the near-peer tutors would need to deliver these sessions effectively? |

**Part C: Closing Remarks**

- Thank the participant for their time.
- Reiterate the confidentiality of their responses.
- Provide contact information in case they have further questions or would like to follow up.
